# The role of uric acid in the insulin resistance in children and adolescents with obesity

**DOI:** 10.1016/j.rpped.2015.03.009

**Published:** 2015

**Authors:** Josiane Aparecida de Miranda, Guilherme Gomide Almeida, Raissa Isabelle Leão Martins, Mariana Botrel Cunha, Vanessa Almeida Belo, José Eduardo Tanus dos Santos, Carlos Alberto Mourão-Júnior, Carla Márcia Moreira Lanna

**Affiliations:** a Universidade Estadual de Campinas (Unicamp), Campinas, SP, Brazil; b Universidade Federal de Juiz de Fora (UFJF), Juiz de Fora, MG, Brazil; c Universidade de São Paulo (USP), Ribeirão Preto, SP, Brazil

**Keywords:** Childhood obesity, Insulin resistance, Uric acid

## Abstract

**Objective:**

To investigate the association between serum uric acid levels and insulin resistance in children and adolescents with obesity.

**Methods:**

Cross-sectional study with 245 children and adolescents (134 obese and 111 controls), aged 8-18 years. The anthropometric variables (weight, height and waist circumference), blood pressure and biochemical parameters were collected. The clinical characteristics of the groups were analyzed by *t*-test or chi-square test. To evaluate the association between uric acid levels and insulin resistance the Pearson's test and logistic regression were applied.

**Results:**

The prevalence of insulin resistance was 26.9%. The anthropometric variables, systolic and diastolic blood pressure and biochemical variables were significantly higher in the obese group (*p*<0.001), except for the high-density-lipoprotein cholesterol. There was a positive and significant correlation between anthropometric variables and uric acid with HOMA-IR in the obese and in the control groups, which was higher in the obese group and in the total sample. The logistic regression model that included age, gender and obesity, showed an odds ratio of uric acid as a variable associated with insulin resistance of 1.91 (95%CI 1.40-2.62; *p*<−0.001).

**Conclusions:**

The increase in serum uric acid showed a positive statistical correlation with insulin resistance and it is associated with and increased risk of insulin resistance in obese children and adolescents.

## Introduction

Uric acid is the end product of purine metabolism, produced by the liver and excreted by the kidneys,^1^ with recognized antioxidant action when its blood levels are within physiological limits.^2^ However, the increase in its serum levels, called hyperuricemia, is an independent risk factor for cardiovascular disease and also has a role in the development of metabolic diseases.^3^^-^ 5 Moreover, recent prospective studies with representative samples indicate hyperuricemia as a predictor for the development of insulin resistance and type 2 diabetes mellitus.^4^^,^ 5 Krishnan et al.^5^ showed that hyperuricemia increases by 1.87-fold the chance of developing type 2 diabetes mellitus and 1.36-fold the chance of developing insulin resistance after 15 years of follow-up.

One of the pathological conditions associated with hyperuricemia is obesity.^6^^,^ 7 Obese individuals have lower renal excretion, and may also have increased production of uric acid.^8^ In children and adolescents, studies indicate that the association between hyperuricemia and obesity is positive^6^ and it is associated with cardiometabolic complications, such as hypertension, atherosclerosis and metabolic syndrome.^9^^-^ 12 Yoo et al.^13^ studied the prevalence of insulin resistance and metabolic syndrome in patients with gout, which is a metabolic disease characterized by hyperuricemia and deposition of monosodium urate crystals in the joints and tissues. The authors concluded that these patients have a higher prevalence of insulin resistance and metabolic syndrome when compared to their healthy peers, and that hyperuricemia in association with insulin resistance may be caused by abdominal obesity.^13^

In this context, in which hyperuricemia has been identified as another link between obesity and insulin resistance, studies in the pediatric population are scarce. Therefore, the aim of this study was to investigate the association between serum uric acid levels and insulin resistance in obese children and adolescents.

## Method

Cross-sectional study with a quantitative approach, part of a project entitled "Association of genetic polymorphisms of cardiovascular relevance with systemic arterial hypertension and obesity in childhood and adolescence", approved by the Institutional Review Board of Universidade Federal de Juiz de Fora, process number 1942.001.2010. All those involved in the research, parents or guardians and children and adolescents were informed of the study objectives and procedures, and those who agreed to participate in the study signed the Informed Consent Form.

The study population consisted of children and adolescents treated at the outpatient clinic of Instituto da Criança e do Adolescente de Juiz de Fora and at the Children's Endocrinology Outpatient Clinic of Núcleo Interdisciplinar de Estudos e Pesquisa em Nefrologia - NIEPEN, of Universidade Federal de Juiz de Fora, Juiz de Fora, Minas Gerais State, Brazil. Convenience sampling was carried out, which included 134 obese children and adolescents in the experimental group and 111 normal-weight children and adolescents. The non-inclusion criteria were chronic diseases, endocrine disorders, use of drugs, signs of acute infection and pregnancy. All children and adolescents underwent physical examination.

Weight and height were measured while participants were wearing light clothing and no shoes. Height was measured with a 0.1 cm-accuracy using a wall stadiometer. Body weight was measured with a digital scale with a 0.1 kg-accuracy. Obesity was defined as body mass index (BMI) above the 95th percentile for age and gender.^14^ Waist circumference (WC) was measured using an inelastic tape at midpoint between the last rib and the upper border of the iliac crest.^15^ Blood pressure was measured by auscultation, and the appropriate cuff size was considered. After resting for 10 min in a quiet environment, blood pressure measurement was taken.^16^ After the physical examination, blood collection was performed by venipuncture in the morning, after 12 h of fasting. The blood was immediately centrifuged at room temperature, and plasma and serum samples were stored at −70 °C until they were analyzed.

The levels of glucose, uric acid and the lipid parameters (total cholesterol, triglycerides and high-density-lipoprotein cholesterol) were measured in serum, with routine enzymatic methods, using commercial kits (Labtest Diagnostics, AS, Lagoa Santa, Brazil). The level of low-density-lipoprotein cholesterol was estimated using the Friedewald formula.^17^ The insulin level was determined by serum enzyme immunoassay kit (Genese Produtos Diagnósticos, São Paulo, Brazil). The estimate of insulin resistance was obtained through the Homeostasis Model Assessment for Insulin Resistance (HOMA-IR), which is the product of fasting insulin (µU/mL) and fasting plasma glucose (mmol/L) divided by 22.5. Insulin resistance is defined when the HOMA-IR value was greater than or equal to 3.16.^18^

Data are shown as mean ± standard deviation. The clinical characteristics of the groups were analyzed using the *t* or Chi-square tests. To evaluate the association between age, BMI, WC, systolic blood pressure, diastolic blood pressure, HDL and uric acid with HOMA-IR, we used Pearson's correlation test. A logistic regression model was also produced to measure the association between uric acid levels (independent variable) and insulin resistance (binary dependent variable). Other independent variables and potentially confounding variables (age, gender and obesity) were included in the regression model in order to control their possible effect on insulin resistance. The selected independent variables were included in the model because of the known association between them and insulin resistance.^6^ The significance level was set at 5% (*p*<0.05) for all tests, and the statistical software used was SPSS version 15.0.

## Results

We evaluated 245 children and adolescents (55.9% females) and the prevalence of insulin resistance was 26.9%. The clinical characteristics of the assessed groups are shown in Table 1. As expected, the obese group had higher BMI and WC values than the control group (*p*<0.001). The systolic and diastolic blood pressures were higher in the obese group when compared to the control group (*p*<0.001). In addition, all the biochemical characteristics, total cholesterol, LDL, triglycerides, glucose, uric acid, insulin and HOMA-IR had higher levels in the obese group, except for the HDL, which was lower in the obese group when compared to the control group (*p*<0.001).

**Table 1:** Clinical characteristics of the study groups.

Variables	Control (*n*=111)	Obese (*n*=134)	*p*
Gender (% F)	66	48	0.005
Age (years)	12.5±2.5	11.2±2.3	<0.001
BMI (kg/m^2^)	18.8±2.6	27.0±4.5	<0.001
WC (cm)	68.7±8.7	90.2±12.8	<0.001
SBP (mmHg)	107.2±10.7	117.9±14.5	<0.001
DBP (mmHg)	66.6±9.2	74.5±9.5	<0.001
Total cholesterol (mg/dL)	134.2±28.6	150.3±40.7	0.001
HDL-c (mg/dL)	44.1±9.7	38.5±9.7	<0.001
LDL-c (mg/dL)	73.7±21.1	91.8±33.1	<0.001
Triglycerides (mg/dL)	74.1±27.1	92.0±44.9	<0.001
Glucose (mg/dL)	82.3±10.5	86.6±9.4	0.001
Insulin (µUI/mL)	9.4±4.0	13.8±6.2	<0.001
HOMA-IR	1.93±0.92	2.98±1.49	<0.001
Uric acid (mg/dL)	3.50±1.12	4.47±1.13	<0.001

F, female; M, male; BMI, body mass index; WC, waist circumference; SBP, systolic blood pressure; DBP, diastolic blood pressure; HDL, high-density lipoprotein; LDL, low-density lipoprotein; HOMA-IR, homeostatic model assessment for insulin resistance.

Table 2 shows the results of the correlation analysis between the variables age, BMI, WC, systolic blood pressure, diastolic blood pressure, HDL and uric acid with HOMA-IR in obese, control groups and in both groups. In the obese group there was a positive and significant correlation between most of the variables with the HOMA-IR, except for age, which was not correlated with HOMA-IR, and HDL, which showed a negative correlation. In the control group, there was a positive and significant correlation between BMI and HOMA-IR and uric acid and HOMA-IR. When analyzing the total sample, there is a positive and significant correlation between most of the variables and the HOMA-IR, except for age, which was not correlated with HOMA-IR, and HDL, which showed a negative correlation. The anthropometric variables (BMI and waist circumference) and uric acid showed the best correlations with HOMA-IR (*r*=0.447, *r*=0.437 and *r*=380; *p*<0.001 respectively).

**Table 2:** Correlation between HOMA-IR and the variables analyzed by groups.

Variables	Obese group			Control group			Total sample	
	HOMA-IR			HOMA-IR			HOMA-IR	
	*r*	*p*		*r*	*p*		*r*	*p*
Age (years)	0.065	0.458		0.036	0.708		−0.075	0.241
BMI (kg/m^2^)	0.355	<0.001		0.239	0.011		0.447	<0.001
SBP (mmHg)	0.261	0.003		0.128	0.197		0.292	<0.001
DBP (mmHg)	0.261	0.003		0.115	0.224		0.256	<0.001
WC (cm)	0.364	<0.001		0.119	0.222		0.437	<0.001
HDL (mg/dL)	−0.118	0.029		−0.027	0.780		−0.258	<0.001
Uric acid (mg/dL)	0.282	0.001		0.214	0.024		0.380	<0.001

BMI, body mass index; WC, waist circumference; SBP, systolic blood pressure; DBP, diastolic blood pressure; HDL, high-density lipoprotein; HOMA-IR, homeostatic model assessment for insulin resistance.

The association of insulin resistance with uric acid levels was also assessed in a logistic regression model, which included age, gender and obesity as independent variables. The logistic regression model produced to measure the association between uric acid levels (independent variable) and insulin resistance (binary dependent variable) included other independent variables and potentially confounding variables (age, gender and obesity), included in the model to control their possible effects on insulin resistance. The dependent variable (insulin resistance) was dichotomized according to the HOMA-IR cutoff point (3.16). The odds ratio (OR) of uric acid was 1.91 (95%CI: 1.40-2.62; *p*<0.001), indicating that the increase of one uric acid unit increases by 91% the likelihood of insulin resistance.

## Discussion

The main finding of this study was the association between serum uric acid levels and insulin resistance in children and adolescents, even after adjusting for age, obesity and gender. After adjusting for these variables, it was observed that, for every increase of 1 mg/dL in serum uric acid levels, there would be a 91% increase in the chance of insulin resistance. Even when analyzing the obese and the control groups alone, uric acid and BMI showed a correlation with HOMA-IR (Table 2).

This finding is in agreement with other authors, who also observed an association between uric acid levels and insulin resistance in studies involving children in a more limited age range.^6^^,^ 9 Gil-Campos et al.^6^ found that in prepubertal obese children, the uric acid levels were significantly higher when compared to the control group, after adjusting for gender, age and BMI associated with insulin resistance. These authors propose that the increase in serum levels of uric acid may be an indicator of early metabolic changes associated with other insulin resistance characteristics. In our study, statistical difference was observed regarding age between the assessed groups, which was only half of the standard deviation (50%), and with normal distribution. Thus, this difference was not considered to be clinically significant, considering the degree of dispersion of this variable.

In fact, in the adult population, recent prospective studies indicate that hyperuricemia is a predictor of insulin resistance and type 2 diabetes mellitus.^4^^,^ 5 ^,^ 19 After a follow-up of 15 years, Krishnan et al.^5^ showed that hyperuricemia increases by 1.87-fold the chance of developing type 2 diabetes mellitus and by 1.36-fold the chance of developing insulin resistance. In a meta-analysis, Kodama et al.^19^ showed an increase of 17% in the risk of type 2 diabetes for every increase of 1 mg/dL in serum uric acid.

Although the physiopathological mechanisms of the connection between hyperuricemia and insulin resistance are not yet clearly established, hyperuricemia is often seen as the result of the reduction in renal excretion of uric acid under the action of hyperinsulinemia.^1^^,^ 20 However, the aforementioned studies oppose this idea, as they show that hyperuricemia precedes insulin resistance.^4^^,^ 5 ^,^ 19

In this study, it was also not possible to answer what the physiopathological mechanisms of serum uric acid contribution on insulin resistance are in obesity, but our work is supported by previous results of studies with animal models and clinical trials, so that some speculations can be made.

Obesity is considered a significant risk factor for the development of insulin resistance and type 2 diabetes mellitus, as in obese individuals the adipose tissue releases substances involved in the development of insulin resistance, such as non-esterified fatty acids, hormones and proinflammatory cytokines.^21^^,^ 22 Adipose tissue has the capacity to secrete cytokines and growth factors that participate in many metabolic processes. Some of these cytokines, with pro-inflammatory characteristics that are increased in obesity, are directly associated with insulin resistance, such as leptin, TNF-α and visfatin, while adiponectin, a cytokine with anti-inflammatory characteristics, is reduced in the presence of obesity and is described as inversely associated with insulin resistance.^21^

Another finding of our study was that the obese group had statistically higher serum uric acid levels when compared to the control group (4.47±1.13 vs. 3.50±1.12, *p*<0.001). This characteristic can be attributed to the following factors: (1) obese individuals have reduced renal clearance of uric acid, which can result in higher serum levels^23^; (2) adipose tissue, similar to the liver and intestine, has abundant activity of xanthine oxidase (enzyme responsible for catalyzing purines and uric acid); and (3) obesity is associated with elevated activity of xanthine oxidase and increased production of uric acid by adipose tissue.^8^

One of the possible links between hyperuricemia and insulin resistance seems to be endothelial dysfunction. Hyperuricemia induction in animals resulted in reduced nitric oxide bioavailability, vasoconstriction and the development of microvascular disease^24^ and, according to Park et al.,^25^ uric acid is responsible for attenuating the production of nitric oxide by reducing the interaction between *eNOS* (endothelial nitric oxide synthase enzyme), and calmodulin. In fact, clinical studies have shown that elevated levels of uric acid are associated with impaired vascular function in children and adolescents.^12^^,^ 26 Thus, the hyperuricemia-mediated endothelial dysfunction could result in lower insulin uptake by reduced blood flow in peripheral tissues (less nitric oxide supply).^1^

In addition to interfering with nitric oxide production, uric acid may also be responsible for its degradation. Although, at physiological concentrations, uric acid has antioxidant effects, thus being an endothelial protective factor, the increase in serum levels causes it to play a pro-oxidant role, as its formation pathway through xanthine oxidase produces reactive oxygen species and hydrogen peroxide, which, in excess, will react with the endothelial nitric oxide and create peroxynitrite, an important oxidizing agent.^27^

Even though the observed mean serum uric acid level in our study was significantly higher in the obese group, when compared with controls, there is no consensus in the literature on the reference values of this marker in children and adolescents, which prevents comparisons. However, we believe that the serum uric acid level is a good alternative to assess cardiometabolic risk even at a young age, as it can be seen in our results. For this purpose, it becomes necessary to carry out studies aimed at establishing reference values to help in clinical diagnosis.

This study has some limitations and, among them, we indicate the use of HOMA-IR to assess insulin resistance. This indicator, although it is not the gold standard, is a widely used method because of its viability. The absence of pubertal status assessment is another limitation, considering the age range used (8-18 years). The cross-sectional nature of the study does not allow us to establish a causal association between the variables.

Based on the observed results, it can be concluded that the increase in serum uric acid levels has a positive statistical correlation with insulin resistance and it is associated with the risk increase of this resistance in obese children and adolescents.
