# Supplementary material for: The role of uric acid in the insulin resistance in children and adolescents with obesity
Source: Rev Paul Pediatr. 2015 Oct-Dec;33(4):431–6. doi: 10.1016/j.rpped.2015.03.009 (PMC4685563; doi:10.1016/j.rpped.2015.03.009)
Supplement: Translation

# Translation: O papel do ácido úrico na resistência insulínica em crianças e adolescentes com obesidade

**Authors:** Josiane Aparecida de Miranda, Guilherme Gomide Almeida, Raissa Isabelle Leão Martins, Mariana Botrel Cunha, Vanessa Almeida Belo, José Eduardo Tanus dos Santos, Carlos Alberto Mourão-Júnior, Carla Márcia Moreira Lanna

**Affiliations:** a Universidade Estadual de Campinas (Unicamp), Campinas, SP, Brasil; b Universidade Federal de Juiz de Fora (UFJF), Juiz de Fora, MG, Brasil; c Universidade de São Paulo (USP), Ribeirão Preto, SP, Brasil

**Keywords:** Obesidade pediátrica, Resistência à insulina, Ácido úrico

## Abstract

**Objetivo:**

Investigar a associação entre os níveis séricos de ácido úrico e a resistência insulínica em crianças e adolescentes com obesidade.

**Métodos:**

Estudo transversal, com 245 crianças e adolescentes (134 obesos e 111 controles), entre oito e 18 anos. Foram coletadas variáveis antropométricas (peso, estatura e circunferência abdominal), pressão arterial e parâmetros bioquímicos. As características clínicas dos grupos foram analisadas pelo teste *t* ou pelo qui-quadrado. Para avaliar a associação entre os níveis de ácido úrico e a resistência insulínica usaram-se o teste de Pearson e regressão logística. A resistência insulínica foi a variável dependente no modelo de regressão.

**Resultados:**

A prevalência de resistência insulínica foi de 26,9%. As variáveis antropométricas, a pressão arterial sistólica e diastólica e as variáveis bioquímicas foram maiores no grupo obeso (*p*<0,001), exceto o colesterol de alta densidade. Foi observada correlação positiva e significativa entre as variáveis antropométricas e o ácido úrico com o HOMA-IR no grupo obeso e no controle. Essa foi maior no grupo obeso e na amostra total. No modelo de regressão logística que incluiu idade, sexo e obesidade, a *odds ratio* do ácido úrico como fator associado à resistência insulínica foi de 1,91 (IC95% 1,40-2,62; *p*<0,001).

**Conclusões:**

Observa-se que o aumento no nível sérico de ácido úrico apresenta correlação estatística positiva com a resistência insulínica e está associado à elevação no risco em crianças e adolescentes obesos.

## Introdução

O ácido úrico é o produto final do metabolismo das purinas, produzido pelo fígado e excretado pelos rins,^1^ com reconhecida ação antioxidante quando seus níveis sanguíneos se encontram dentro dos limites fisiológicos.^2^ Porém, a elevação nos níveis séricos, denominada hiperuricemia, é considerada fator de risco independente para doenças cardiovasculares e também tem papel no desenvolvimento de doenças metabólicas.^3^^-^ 5 Além disso, estudos prospectivos recentes, com amostras representativas, apontam a hiperuricemia como preditora do desenvolvimento de resistência insulínica e da diabetes melito tipo 2.^4^^,^ 5 Krishnan et al.^5^demonstraram que a hiperuricemia aumenta em 1,87 vez a chance de desenvolver diabetes melito tipo 2 e em 1,36 vez a chance de desenvolver resistência insulínica após 15 anos de seguimento.

Uma das condições patológicas associadas à hiperuricemia é a obesidade.^6^^,^ 7 Indivíduos obesos mostram menor excreção renal de ácido úrico e podem apresentar também maior produção.^8^ Em crianças e adolescentes, estudos apontam que a relação entre hiperuricemia e obesidade é positiva^6^ e associada a complicações cardiometabólicas como hipertensão, aterosclerose e síndrome metabólica.^9^^-^ 12 Yoo et al.^13^ avaliaram a prevalência de resistência insulínica e síndrome metabólica em pacientes com gota, que é uma doença metabólica caracterizada por hiperuricemia e deposição de cristais de monourato de sódio nas articulações e nos tecidos. Os autores concluíram que esses pacientes têm maior prevalência de resistência insulínica e síndrome metabólica, comparados com seus pares saudáveis, e que a hiperuricemia, em associação com a resistência insulínica, pode ter como causa a obesidade abdominal.^13^

Nesse contexto, em que a hiperuricemia tem sido apontada como mais um elo entre a obesidade e a resistência insulínica, estudos com a população pediátrica ainda são escassos. Portanto, o objetivo deste estudo foi investigar a associação entre os níveis séricos de ácido úrico e resistência insulínica em crianças e adolescentes com obesidade.

## Método

Estudo de delineamento transversal, com abordagem quantitativa, parte de um projeto intitulado "Associação de polimorfismos genéticos de relevância cardiovascular com hipertensão arterial sistêmica e obesidade na infância e adolescência" aprovado pelo Comitê de Ética em Pesquisa da Universidade Federal de Juiz de Fora, MG, Brasil, sob o número 1942.001.2010. Todos os envolvidos na pesquisa, pais ou responsáveis e crianças e adolescentes, foram esclarecidos sobre os objetivos e procedimentos da pesquisa e os que concordaram em participar assinaram o Termo de Consentimento Livre e Esclarecido.

A população deste estudo foi constituída por crianças e adolescentes atendidas no ambulatório no Instituto da Criança e do Adolescente de Juiz de Fora e no Ambulatório de Endocrinologia Infantil do Núcleo Interdisciplinar de Estudos e Pesquisa em Nefrologia (Niepen) da Universidade Federal de Juiz de Fora. Foi feita uma amostragem por conveniência, que incluiu 134 crianças e adolescentes obesos no grupo experimental e 111 crianças e adolescentes eutróficos. Os critérios de não inclusão foram doença crônica, doenças endócrinas, uso de fármacos, sinais de infecção aguda e gravidez. Todas as crianças e todos os adolescentes foram submetidos a exame físico.

O peso e altura foram medidos com os participantes vestindo roupas leves e sem calçados. A altura foi medida com precisão de 0,1cm, com -se um estadiômetro de parede. O peso corporal foi medido com uma balança digital de precisão de 0,1kg. A obesidade foi definida como índice de massa corporal (IMC) acima do percentil 95 de acordo com idade e sexo.^14^ A circunferência abdominal (CA) foi medida com fita inelástica, no ponto médio entre a última costela e a borda superior da crista ilíaca.^15^ A pressão arterial foi aferida pelo método auscultatório e considerados o tamanho adequado do manguito. Após repouso de 10 minutos em ambiente calmo, foi feita a aferição da pressão arterial.^16^ Após o exame físico, foi feita a coleta de sangue, por punção venosa de manhã, depois de 12 horas de jejum. O sangue foi imediatamente centrifugado à temperatura ambiente e as amostras de plasma e soro foram armazenadas a -70 °C até ser analisadas.

As concentrações de glicose e ácido úrico e os parâmetros lipídicos (colesterol total, triglicérides e lipoproteína de alta densidade [HDL]) foram determinados em soro, com métodos enzimáticos de rotina, com o uso de kits comerciais (Labtest Diagnósticos, AS, Lagoa Santa, Brasil). A concentração da lipoproteína de baixa densidade (LDL) foi estimada pela fórmula de Friedewald.^17^ A concentração de insulina foi determinada por imunoensaio enzimático em soro (kit Genese Produtos Diagnósticos, São Paulo, Brasil). A estimativa da resistência à insulina foi obtida por meio do Homeostasis Model Assessment for Insulin Resistance (HOMA-IR), que é o produto da insulina de jejum (µU/mL) e da glicemia de jejum (mmol/L) dividido por 22,5. A resistência à insulina foi definida quando o valor de HOMA-IR foi maior ou igual a 3,16.^18^

Os dados estão apresentados como média ± desvio padrão. As características clínicas dos grupos foram analisadas por meio do teste *t* ou qui-quadrado. Para avaliar a associação entre idade, IMC, CA, pressão arterial sistólica, pressão arterial diastólica, HDL e ácido úrico com o HOMA-IR, usamos o teste de correlação de Pearson*.* Foi também produzido um modelo de regressão logística para mensurar a associação entre os níveis de ácido úrico (variável independente) e a resistência insulínica (variável dependente binária). Outras variáveis independentes e potenciais variáveis de confusão (idade, sexo e obesidade) foram incluídas no modelo de regressão com a finalidade de controlar seu possível efeito na resistência insulínica. As variáveis independentes selecionadas foram incluídas no modelo em virtude da conhecida associação entre elas e a resistência insulínica.^6^ O nível de significância adotado foi de 5% (*p*<0,05) para todos os testes e o programa estatístico usado foi o SPSS versão 15.0.

## Resultados

Avaliamos 245 crianças e adolescentes (55,9% do sexo feminino) e a prevalência de resistência insulínica foi de 26,9%. As características clínicas dos grupos avaliados estão representadas na Tabela 1. Como esperado, o grupo obeso apresentou IMC e CA maiores do que o grupo controle (*p*<0,001). A pressão arterial sistólica e a diastólica foram mais elevadas no grupo obeso, comparado com o grupo controle (*p*<0,001). Além disso, todas as características bioquímicas, colesterol total, LDL, triglicérides, glicose, ácido úrico, insulina e HOMA-IR apresentaram-se em níveis mais altos no grupo obeso, exceto o HDL, que foi mais baixo no grupo obeso comparado com o grupo controle (*p*<0,001).

**Tabela 1:** Características clínicas dos grupos estudados

Variáveis	Controle (n=111)	Obeso (n=134)	*p*
Gênero (% F)	66	48	0,005
Idade (anos)	12,5±2,5	11,2±2,3	<0,001
IMC (Kg/m^2^)	18,8±2,6	27,0±4,5	<0,001
CA (cm)	68,7±8,7	90,2±12,8	<0,001
PAS (mmHg)	107,2±10,7	117,9±14,5	<0,001
PAD (mmHg)	66,6±9,2	74,5±9,5	<0,001
Colesterol total (mg/dL)	134,2±28,6	150,3±40,7	0,001
HDL‐c (mg/dL)	44,1±9,7	38,5±9,7	<0,001
LDL‐c (mg/dLl)	73,7±21,1	91,8±33,1	<0,001
Triglicerídeos (mg/dL)	74,1±27,1	92,0±44,9	<0,001
Glicose (mg/dL)	82,3±10,5	86,6±9,4	0,001
Insulina (µUI/mL)	9,4±4,0	13,8±6,2	<0,001
HOMA–IR	1,93±0,92	2,98±1,49	<0,001
Ácido úrico (mg/dL)	3,50±1,12	4,47±1,13	<0,001

F, feminino; M, masculino; IMC, índice de massa corporal; CA, circunferência abdominal; PAS, pressão arterial sistólica; PAD, pressão arterial diastólica; HDL, lipoproteína de alta densidade; LDL, lipoproteína de baixa densidade; HOMA‐IR, modelo de avaliação da homeostase‐índice de resistência insulínica.

Na Tabela 2 estão apresentados os resultados da análise de correlação entre as variáveis idade, IMC, CA, pressão arterial sistólica, pressão arterial diastólica, HDL e ácido úrico com o HOMA-IR nos grupos obeso, controle e em ambos os grupos. No grupo obeso foi observada correlação positiva e significativa entre a maioria das variáveis com o HOMA-IR, exceto a idade, que não se correlacionou com o HOMA-IR, e o HDL, que apresentou correlação negativa. No grupo controle, foi observada correlação positiva e significativa entre o IMC e o HOMA-IR e o ácido úrico e o HOMA-IR. Quando analisada a amostra total, observa-se correlação positiva e significativa entre a maioria das variáveis e o HOMA-IR, exceto a idade, que não se correlacionou com o HOMA-IR, e o HDL, que apresentou correlação negativa. As variáveis antropométricas (IMC e circunferência abdominal) e o ácido úrico foram as que apresentaram melhor correlação com o HOMA-IR (r=0,447, r=0,437 e r=380; *p*<0,001 respectivamente).

**Tabela 2:** Correlação entre HOMA‐IR e as variáveis analisadas por grupos

		Grupo Obeso		Grupo Controle			Amostra total	
Variáveis		HOMA‐IR		HOMA‐IR			HOMA‐IR	
	r	*p*		r	*p*		r	*p*
Idade (anos)	0,065	0,458		0,036	0,708		‐0,075	0,241
IMC (kg/m^2^)	0,355	<0,001		0,239	0,011		0,447	<0,001
PAS (mmHg)	0,261	0,003		0,128	0,197		0,292	<0,001
PAD (mmHg)	0,261	0,003		0,115	0,224		0,256	<0,001
CA (cm)	0,364	<0,001		0,119	0,222		0,437	<0,001
HDL (mg/dL)	‐0,118	0,029		‐0,027	0,780		‐0,258	<0,001
Ácido úrico (mg/dL)	0,282	0,001		0,214	0,024		0,380	<0,001

IMC, índice de massa corporal; CA, circunferência abdominal; PAS, pressão arterial sistólica; PAD, pressão arterial diastólica; HDL, lipoproteína de alta densidade; HOMA‐IR, modelo de avaliação da homeostase‐índice de resistência insulínica.

Analisou-se ainda a associação de resistência insulínica com os níveis de ácido úrico, em um modelo de regressão logística que incluiu idade, sexo e obesidade como variáveis independentes. O modelo de regressão logística produzido para mensurar a associação entre os níveis de ácido úrico (variável independente) e a resistência insulínica (variável dependente binária) incluiu outras variáveis independentes e potenciais variáveis de confusão (idade, sexo e obesidade), inseridas no modelo com a finalidade de controlar seus possíveis efeitos na resistência insulínica. A variável dependente (resistência insulínica) foi dicotomizada em função do ponto de corte do HOMA-IR (3,16). O *odds ratio* (OR) do ácido úrico foi 1,91 (IC95%: 1,40 a 2,62; *p*<0,001). Isso indica que o aumento de uma unidade ácido úrico eleva em 91% a probabilidade de resistência insulínica.

## Discussão

O principal achado deste estudo foi a associação entre os níveis séricos de ácido úrico e a resistência insulínica em crianças e adolescentes, mesmo após os ajustes para idade, obesidade e sexo. Depois do ajuste para essas variáveis, observou-se que a cada aumento de 1 mg/dL nos níveis séricos de ácido úrico haveria um aumento de 91% na chance de resistência insulínica. Mesmo ao analisar o grupo obeso e o controle de forma isolada, o ácido úrico e o IMC mostraram correlação com o HOMA-IR (Tabela 2).

Esse achado está de acordo com outros autores que também observaram associação entre os níveis de ácido úrico e resistência insulínica em estudos que envolveram crianças em faixa etária mais restrita.^6^^,^ 9Gil-Campos et al.^6^ verificaram que em crianças obesas pré-puberes a concentração de ácido úrico foi significativamente maior, comparada com o grupo controle, após ajuste para sexo, idade e IMC associada à resistência insulínica. Esses autores propõem que a elevação sérica do ácido úrico pode ser um indicador de alteração metabólica precoce associada com outras características da resistência à insulina. Em nosso estudo, observou-se diferença estatística na idade entre os grupos avaliados, que foi de apenas metade do desvio padrão (50%), e com distribuição normal. Assim, considerou-se que a diferença não foi clinicamente relevante, tendo em vista o grau de dispersão dessa variável.

De fato, na população adulta, estudos prospectivos recentes apontam ser a hiperuricemia preditora de resistência insulínica e diabetes melito tipo 2.^4^^,^ 5 ^,^ 19 Após seguimento de 15 anos, Krishnan et al.^5^evidenciaram que a hiperuricemia aumenta em 1,87 vez a chance de desenvolver diabetes melito tipo 2 e em 1,36 vez a chance de desenvolver resistência insulínica. Em metanálise, Kodama et al.^19^ apontaram aumento de 17% no risco de diabete tipo 2 para cada aumento de 1 mg/dL no ácido úrico sérico.

Embora os mecanismos fisiopatológicos da ligação entre a hiperuricemia e a resistência insulínica ainda não estejam claramente estabelecidos, a hiperuricemia é, com frequência, apontada como o resultado da redução na excreção renal de ácido úrico sob ação da hiperinsulinemia.^1^^,^ 20Todavia, os estudos citados se contrapõem a essa ideia por evidenciar que a hiperuricemia precede a resistência insulínica.^4^^,^ 5 ^,^ 19

No presente estudo, também não foi possível responder quais são os mecanismos fisiopatológicos da contribuição dos níveis séricos de ácido úrico sobre a resistência insulínica na obesidade, mas nos apoiaremos em resultados anteriores, tanto de estudos com modelos animais como de estudos clínicos, para fazer algumas especulações.

A obesidade é considerada importante fator de risco para a resistência insulínica e desenvolvimento de diabetes melito tipo 2, uma vez que, em indivíduos obesos, o tecido adiposo libera substâncias envolvidas no desenvolvimento de resistência à insulina, como ácidos graxos não esterificados, hormônios e citocinas pró-inflamatórias.^21^^,^ 22 O tecido adiposo tem capacidade de secretar citocinas e fatores de crescimento que participam de diversos processos metabólicos. Algumas dessas citocinas, com características pró-inflamatórias e aumentadas na obesidade, estão diretamente associadas à resistência a insulina, como a leptina, o TNF-α e a visfatina, enquanto a adiponectina, uma citocina com característica anti-inflamatória, está reduzida na presença de obesidade e é descrita como inversamente associada à resistência insulínica.^21^

Outro achado do nosso estudo foi que o grupo obeso apresentou níveis séricos de ácido úrico estatisticamente mais elevados, comparado com o grupo controle (4,47±1,13 *vs.* 3,50±1,12, *p*<0,001). Essa característica pode ser atribuída aos seguintes fatores: 1) indivíduos obesos apresentam redução na depuração renal de ácido úrico, o que pode implicar maiores níveis séricos;^23^ 2) o tecido adiposo, similar ao fígado e ao intestino, apresenta abundante atividade da xantina oxidase (enzima responsável por catalisar purinas a ácido úrico) e a obesidade é associada com elevada atividade da xantina oxidase e maior produção de ácido úrico pelo tecido adiposo.^8^

Um dos possíveis elos entre a hiperuricemia e a resistência insulínica parece ser a disfunção endotelial. A indução de hiperuricemia em animais resultou em redução na biodisponibilidade de óxido nítrico, vasoconstrição e desenvolvimento de doença microvascular^24^ e, segundo Park et al.,^25^ o ácido úrico é responsável por atenuar a produção de óxido nítrico por meio da diminuição da interação entre a *eNOS* (enzima óxido nítrico sintase endotelial) e a calmodulina. De fato, estudos clínicos apontam que elevados níveis de ácido úrico estão associados a prejuízo na função vascular de crianças e adolescentes^12^^,^ 26 Assim, a disfunção endotelial mediada pela hiperuricemia poderia resultar em menor captação de insulina pela redução no fluxo sanguíneo em tecidos periféricos (menor oferta de óxido nítrico).^1^

Além de interferir na produção de óxido nítrico, o ácido úrico também pode ser responsável pela sua degradação. Embora, em concentrações fisiológicas, o ácido úrico tenha efeito antioxidante e seja, portanto, um fator de proteção endotelial, a elevação nos níveis séricos faz com que assuma um papel pró-oxidante, pois sua via de formação pela xantina oxidase produz espécies reativas de oxigênio e peróxido de hidrogênio, que, em excesso, irão reagir com o óxido nítrico endotelial e formar o peroxinitrito, importante agente oxidante.^27^

Embora a concentração sérica média de ácido úrico observada em nosso estudo tenha se mostrado significativamente maior no grupo obeso comparado com o controle, não existe concordância na literatura sobre os valores de referência desse marcador em crianças e adolescentes, o que impede comparações. Todavia, acreditamos que a dosagem do ácido úrico sérico seja uma boa opção para avaliar o risco cardiometabólico mesmo em faixa etária jovem, como é possível constatar em nossos resultados. Para isso, tornam-se necessários estudos que visem a estabelecer valores de referência para auxiliar no diagnóstico clínico.

Este estudo apresenta algumas limitações e, dentre elas, aponta-se o uso do HOMA-IR para avaliar a resistência insulínica. Esse indicador, embora não seja o padrão ouro, é um método amplamente usado devido à sua viabilidade. A ausência de avaliação do estado puberal é outra limitação deste estudo, tendo em vista a variação da faixa etária analisada (oito a 18 anos). A natureza transversal do estudo também não nos permite estabelecer relação de causalidade entre as variáveis.

Com base nos resultados observados, pode-se concluir que o aumento no nível sérico de ácido úrico apresenta correlação estatística positiva com a resistência insulínica e se associa à elevação no risco em crianças e adolescentes obesos.
